# Experimental genetic crosses in tsetse flies of the livestock pathogen *Trypanosoma congolense* savannah

**DOI:** 10.1186/s13071-023-06105-4

**Published:** 2024-01-04

**Authors:** Lori Peacock, Chris Kay, Mick Bailey, Wendy Gibson

**Affiliations:** 1 School of Biological Sciences, University of Bristol, Bristol, BS8 1TQ UK; 2 Bristol Veterinary School, University of Bristol, Langford, Bristol, BS40 7DU UK

**Keywords:** *Trypanosoma congolense*, Sexual reproduction, Tsetse fly, Mating, Green fluorescent protein, Red fluorescent protein

## Abstract

**Background:**

In tropical Africa animal trypanosomiasis is a disease that has severe impacts on the health and productivity of livestock in tsetse fly-infested regions. *Trypanosoma congolense* savannah (*TCS*) is one of the main causative agents and is widely distributed across the sub-Saharan tsetse belt. Population genetics analysis has shown that *TCS* is genetically heterogeneous and there is evidence for genetic exchange, but to date *Trypanosoma brucei* is the only tsetse-transmitted trypanosome with experimentally proven capability to undergo sexual reproduction, with meiosis and production of haploid gametes. In *T. brucei* sex occurs in the fly salivary glands, so by analogy, sex in *TCS* should occur in the proboscis, where the corresponding portion of the developmental cycle takes place. Here we test this prediction using genetically modified red and green fluorescent clones of *TCS*.

**Methods:**

Three fly-transmissible strains of *TCS* were transfected with genes for red or green fluorescent protein, linked to a gene for resistance to the antibiotic hygromycin, and experimental crosses were set up by co-transmitting red and green fluorescent lines in different combinations via tsetse flies, *Glossina pallidipes*. To test whether sex occurred in vitro, co-cultures of attached epimastigotes of one red and one green fluorescent *TCS* strain were set up and sampled at intervals for 28 days.

**Results:**

All interclonal crosses of genetically modified trypanosomes produced hybrids containing both red and green fluorescent proteins, but yellow fluorescent hybrids were only present among trypanosomes from the fly proboscis, not from the midgut or proventriculus. It was not possible to identify the precise life cycle stage that undergoes mating, but it is probably attached epimastigotes in the food canal of the proboscis. Yellow hybrids were seen as early as 14 days post-infection. One intraclonal cross in tsetse and in vitro co-cultures of epimastigotes also produced yellow hybrids in small numbers. The hybrid nature of the yellow fluorescent trypanosomes observed was not confirmed by genetic analysis.

**Conclusions:**

Despite absence of genetic characterisation of hybrid trypanosomes, the fact that these were produced only in the proboscis and in several independent crosses suggests that they are products of mating rather than cell fusion. The three-way strain compatibility observed is similar to that demonstrated previously for *T. brucei*, indicating that a simple two mating type system does not apply for either trypanosome species.

**Graphical Abstract:**

**Figure:**
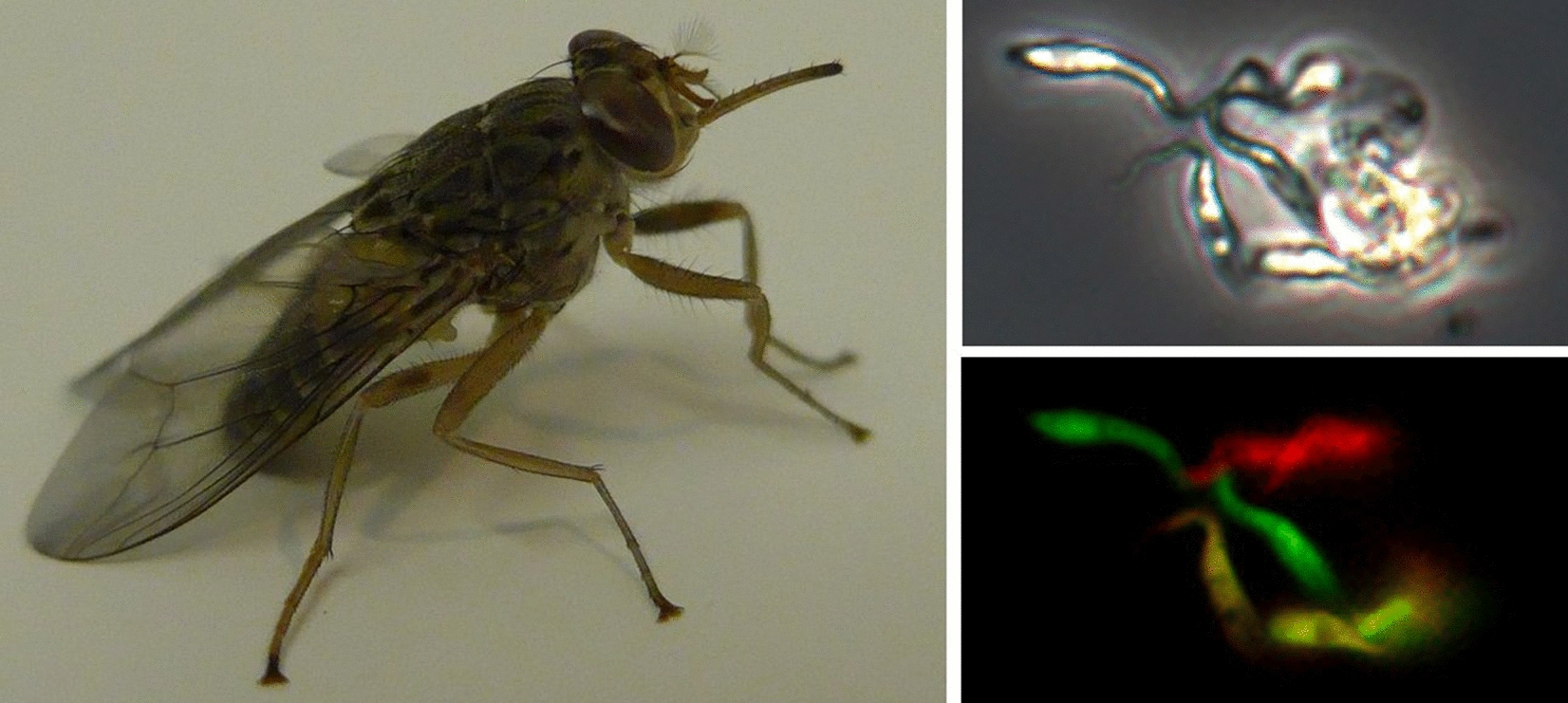

**Supplementary Information:**

The online version contains supplementary material available at 10.1186/s13071-023-06105-4.

## Background

In tropical Africa animal trypanosomiasis (AAT, nagana) is a disease that severely impacts on the health and productivity of livestock in the tsetse fly-infested regions, which occupy > 9 million km^2^ or one third of Africa’s land mass [1]. AAT is a major problem in terms of veterinary health and agricultural productivity and hence has large negative economic consequences [2–4]. For example, an estimated 50 million head of cattle are at risk of infection, with an estimated 3 million deaths despite 35 million doses of trypanocidal drugs administered per year [5]. Besides cattle, trypanosomiasis affects sheep, goats, pigs, horses, donkeys and camels kept in the tsetse belt. In 2012, production losses due to cattle trypanosomiasis in Africa were estimated at US$ 1.0–1.2 billion per year [1], while AU-IBAR estimated far greater losses in agricultural production at US$ 4.5 billion per year [5]. Losses arise in livestock productivity (meat, milk, breeding stock, fertility, traction power, transport, manure) and have long-term impact in lost agricultural potential.

AAT is caused by parasitic protozoa of the genus *Trypanosoma*, which are motile single-celled organisms found in the blood of the mammalian host and in the gut and mouthparts of the tsetse fly (genus *Glossina*). *Trypanosoma congolense*, *T. vivax* and *T. brucei* are recognised as the main agents of AAT. Each of these species is subdivided into different genotypes; for example, *T. congolense* is not a single species but comprises three genetically distinct subgroups, savannah (*TCS*), forest (*TCF*) and kilifi or Kenya coast (*TCK*) [6], which occur in overlapping host and geographic distributions. *TCS* is considered the most widely prevalent and pathogenic *T. congolense* subgroup [7, 8]. Sexual reproduction is now well characterized in *T. brucei*, including the description of meiosis and haploid gametes [9–12]. *TCS* is also believed to undergo genetic exchange, based on population genetics analysis by microsatellites of field-collected isolates from The Gambia [13] and evidence of putative hybrids from whole genome sequencing of isolates from Zambia [8]; however, no one has demonstrated the process experimentally so far. By analogy with *T. brucei*, for which sexual reproduction occurs in the tsetse salivary glands [9], mating in *TCS* is expected to occur in the tsetse fly vector among the stages that develop in the proboscis. However, a previous, detailed study of the *TCS* life cycle [14] failed to identify meiotic division stages or gametes resembling those found in *T. brucei* [10, 11], although we have recently identified these stages by morphology in the fly proboscis for the related species *T. simiae* [15], which has a similar developmental cycle to *TCS*. Importantly, if *TCS* is capable of sexual reproduction, genes for harmful traits, such as drug resistance or virulence, could rapidly spread among strains of this livestock pathogen in the field.

We previously used genetically modified red and green fluorescent trypanosome lines to study sexual reproduction in *T. brucei* [9, 16, 17] and here adapted this successful approach to discover whether mating occurs in *TCS*. In this system, hybrids that inherit the transgenes for both red and green fluorescence appear yellow, making them easy to distinguish from the parental red and green fluorescent trypanosomes. A further advantage of the red/green cross design is that tsetse flies containing a mixture of the two parental strains, and therefore most likely to contain mating trypanosomes, are easily identified.

## Methods

### Trypanosomes

Three fly-transmissible strains of *T. congolense* savannah (*TCS*) were used (Table 1). Trypanosomes were grown as free-swimming procyclics or attached epimastigotes in Cunningham’s medium (CM) [18] supplemented with 10 µg/ml gentamycin, 5 µg/ml hemin and 15% heat-inactivated foetal calf serum (FCS) at 27 °C [19]. Epimastigotes were grown as attached cell layers in T25 flasks (Nunclon) laid flat or in 24-well plates (Nunclon) containing a sterile round (12-mm-diameter) coverslip; the overlay was replaced every few days. For the in vitro mixing experiment, clumps of cells from each epimastigote culture were dislodged, mixed in approximately equal numbers and transferred to individual 1-ml wells, allowing the co-culture to be destructively sampled at intervals (*T* = 1, 3, 5, 6, 21, 28 days). The three *TCS* strains were genotyped by microsatellite analysis [13]; microsatellite loci TCM3 and TCM6 discriminated well among all three strains.

**Table 1:** *Trypanosoma congolense* savannah (*TCS*) strains used in experimental crosses

*TCS* strain	Host	Location	Year of isolation
WG81	Goat	Matuga, Kenya	1981
Gam2	Bovine	Keneba, The Gambia	1977
1/148	Bovine	Nigeria	1960

### Transfection

Plasmid constructs suitable for stable transfection of *TCS* were modified from pHD67E [20] by replacement of the *T. brucei*-specific targeting region with the analogous ribosomal RNA (rRNA) non-transcribed spacer from *TCS*; this region was PCR-amplified from Gam2 genomic DNA and contained a central NotI site for linearisation. A *TCS*-specific rRNA promotor followed by the 5′ UTR from *GARP* (from pPROMOEXP [21]) was inserted between the PARP promotor and *eGFP* gene to create plasmid pHOG. Replacement of *eGFP* with the *mRFP* gene yielded plasmid pHOR. The fluorescent protein genes had a *T. brucei* aldolase 3′ UTR and the downstream hygromycin resistance gene was flanked by *T. brucei* actin 5′ and 3′ UTRs. Each transfection was done by electroporation (Amaxa Nucleofector 2B program X-001) using ~ 5 × 10^7^ trypanosomes in late log growth and 5–10 µg of plasmid DNA [19]. Selection with hygromycin (25 µg/ml) commenced 24 h later and cultures were checked for growth of fluorescent cells after a week. Once successfully transfected cultures were growing strongly, trypanosomes were cloned by limiting dilution. Transfected lines were fly transmitted to check that they maintained ability to develop into metacyclics.

### Tsetse flies and dissection

*Glossina pallidipes* tsetse flies were kept at 25 °C and 70% relative humidity and fed on sterile defibrinated horse blood supplemented with 1 mM dATP [22] via a silicone membrane. Flies were given an infective blood meal for their first feed 24–48 h post-eclosion. The infective blood meal contained approximately equal numbers (~ 10^7^ cells/ml) of procyclic or epimastigote form trypanosomes of each of two strains in CM mixed with an equal volume of washed horse red blood cells resuspended in Hank’s Balanced Salt Solution, supplemented with 10 mM l-glutathione to increase infection rates [23]. *TCS* strain Gam2 was outcompeted by 1/148, so for this combination the ratio of Gam2 to 1/148 was increased two to five fold in the infective blood meal. All possible combinations of red and green fluorescent strains were mixed in interclonal crosses; in addition, an intraclonal cross of 1/148 was tried.

Flies were dissected 14–40 days after infection. Whole tsetse alimentary tracts were dissected from the abdomen; the proventriculus and remainder of the midgut were placed into separate drops of phosphate-buffered saline (PBS) and the presence and colour of trypanosomes was recorded. Proboscides were dissected into a separate drop of PBS and teased apart with forceps and fine needles to separate mouthparts and aid release of trypanosomes. Trypanosome-infected proboscides were identified by phase contrast microscopy, but intense autofluorescence of the proboscis made it difficult to determine the colour of fluorescent trypanosomes. Two techniques were used to overcome this problem: (i) viewing trypanosomes released from the proboscis rather than those attached inside or within the hypopharynx, (ii) incubating proboscides in PBS supplemented with 20% FCS and × 1 Anti-contamination cocktail (ACC; [24]) in a 96-well plate at 27 °C for 4–5 days to allow trypanosomes to increase in number before proboscides were removed and teased apart.

### Imaging

Samples were viewed using a DMRB microscope (Leica) equipped with a Retiga Exi camera (QImaging) and Volocity software (PerkinElmer). To view nuclei and kinetoplasts, live trypanosomes were stained with Hoechst 33342 as follows: dissected proboscides were pooled and left for about 15 min to allow trypanosomes to spill out before the fly tissue was removed and the trypanosomes washed once with 100 µl PBS by centrifugation at 3000 rpm for 5 min at room temperature (RT); trypanosomes were resuspended in the residual PBS (~ 15 µl) before addition of 2 µl of a 1:100 dilution of Hoechst 33342 in PBS; after incubation in the dark for 15 min at RT, the preparation was placed on a microscope slide under a coverslip and viewed immediately by fluorescence microscopy.

## Results

### Trypanosome transfection

Three fly-transmissible *TCS* strains, WG81, Gam2 and 1/148 (Table 1), were each transfected with a plasmid construct containing the gene for either red or green fluorescent protein, linked to a gene for resistance to the antibiotic hygromycin (plasmids pHOR and pHOG respectively). Transfected procyclic cultures were selected by antibiotic resistance and subsequently the transgenic cell lines were cloned and fly transmitted to verify that they maintained the ability to complete the full life cycle and stably maintain expression of the transgene. This provided a panel of potentially compatible *TCS* parental lines to test in experimental crosses. Previous experience showed that the identification of mating-compatible strains was a key factor for success of *T. brucei* crosses.

### Trypanosome crosses

**Fig. 1:**
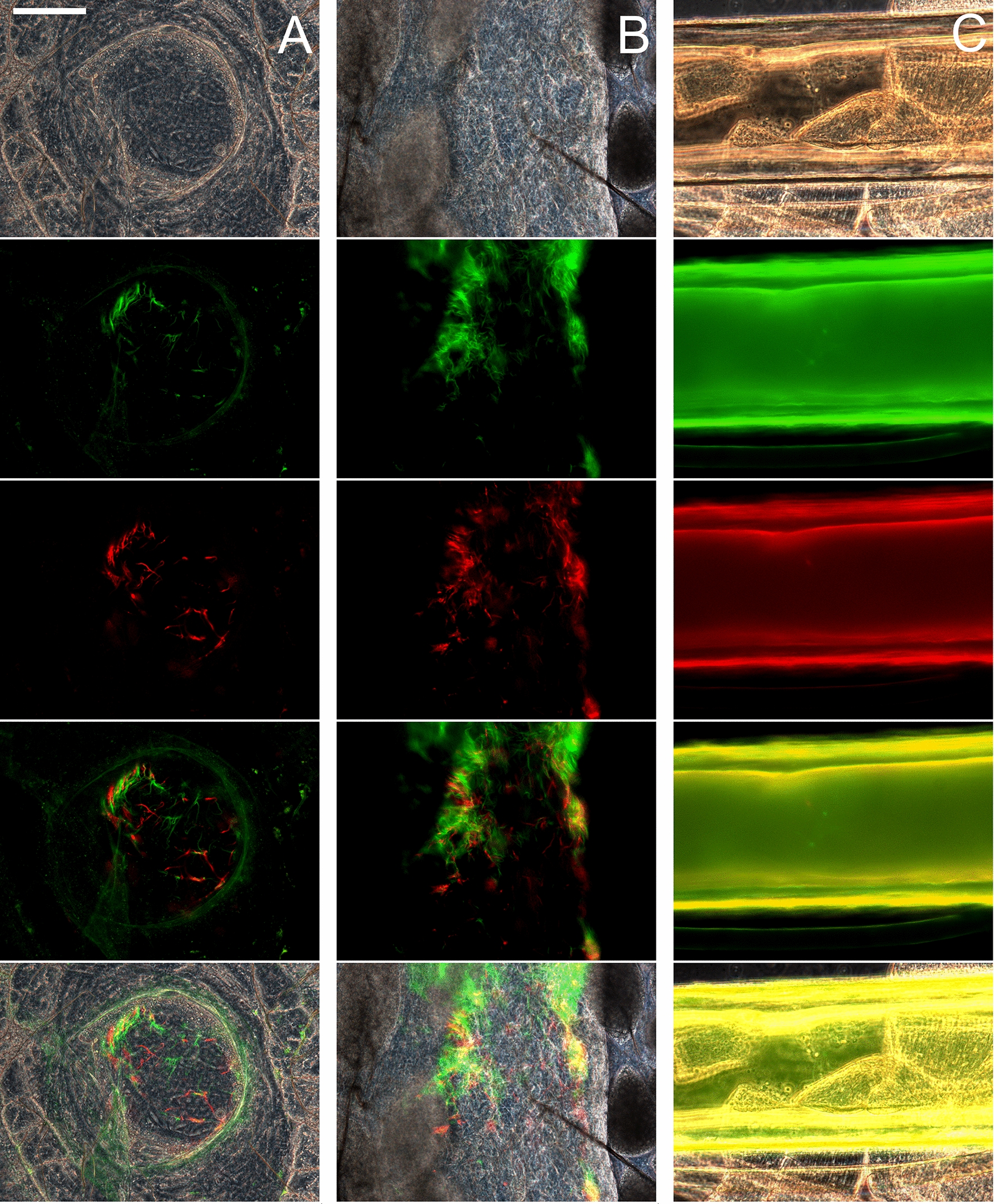
Visualization of fluorescent trypanosomes in tsetse fly organs. Red and green fluorescent *Trypanosoma congolense* savannah (*TCS*) in proventriculus (**A**) and midgut (**B**) from a tsetse fly dissected 25 days post-infection with a mixture of Gam2 RFP and 1/148 GFP. **C** Autofluorescence of the tsetse proboscis; a portion of an uninfected labrum is shown. Rows top to bottom: phase contrast, green fluorescence, red fluorescence, merge of red and green fluorescence, merge all. Areas of overlapping red and green fluorescence appear yellow in the merged images of the proventriculus (**A**) and midgut (**B**), but no yellow fluorescent trypanosomes were seen when the preparations were squashed under the coverslip to separate individual trypanosomes. Scale bar = 50 µm

**Fig. 2:**
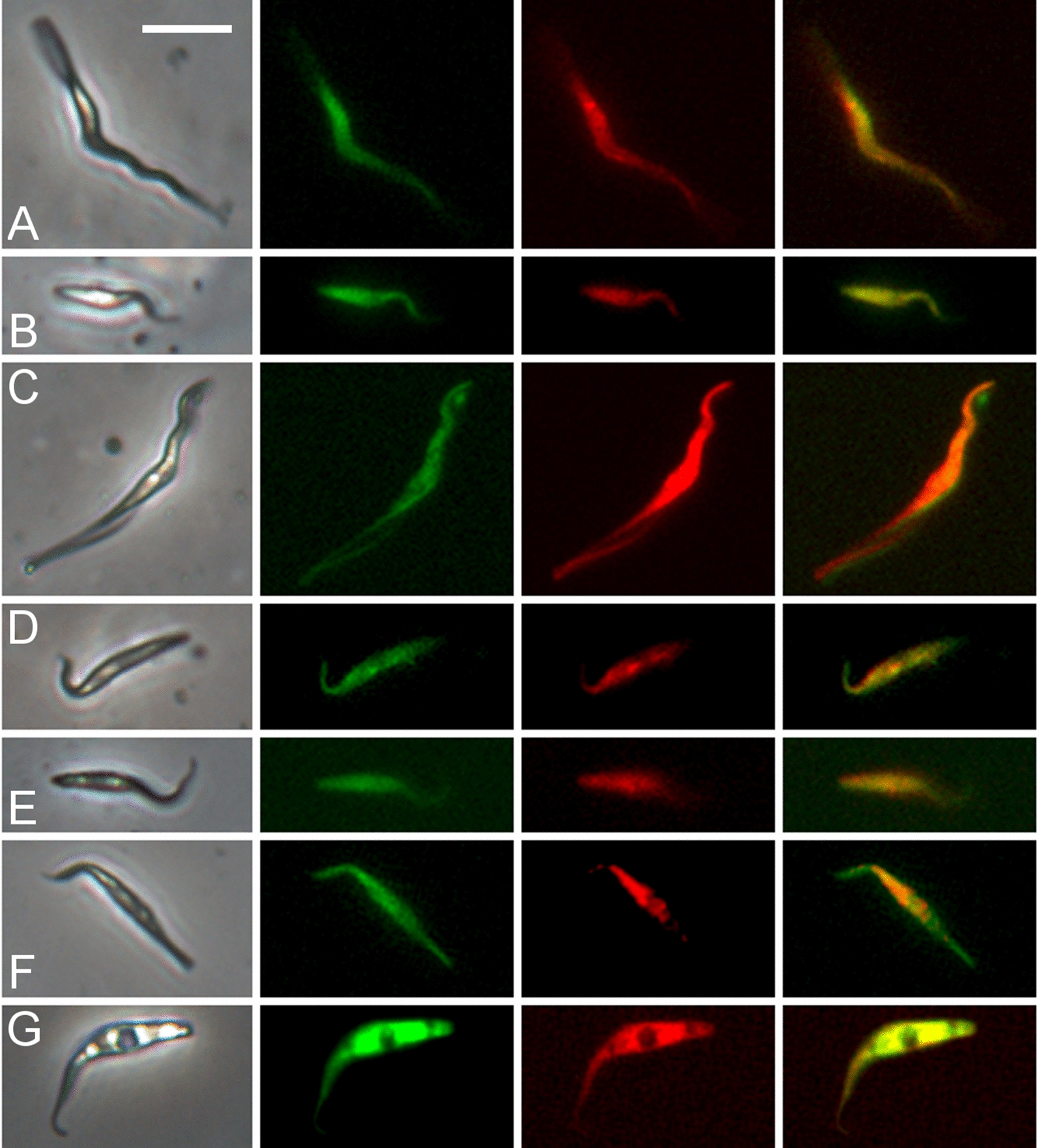
Examples of yellow fluorescent trypanosomes from proboscides of flies infected with mixtures of different *TCS* strains. Flies were dissected 23–39 days post-infection. **A**,** B** Gam2 RFP × 1/148 GFP; **C**,** D** 1/148 RFP × WG81 GFP; **E**, **F** Gam2 RFP × WG81 GFP; **G** intraclonal cross 1/148 RFP × 1/148 GFP. From left to right: phase contrast, green fluorescence, red fluorescence, merge of red and green fluorescence. Scale bar = 10 µm

Crosses were set up by co-infecting tsetse flies (*G. pallidipes*) with pairs of red and green fluorescent trypanosome lines and flies were dissected 14–40 days after the infected feed. In tsetse, *TCS* first colonises the midgut followed by anterior migration to the proboscis via the proventriculus and foregut [14]. Most midgut and proventricular populations contained both red and green fluorescent trypanosomes, but no yellow fluorescent trypanosomes were seen (Fig. 1A, B). However, yellow fluorescent trypanosomes were present among trypanosomes isolated from the proboscis. Intense autofluorescence of the proboscis (Fig. 1C) obstructed the direct observation of any fluorescent trypanosomes within, and therefore fluorescence colour was recorded from trypanosomes released from the proboscis and not from those attached to the inner wall or inside the hypopharynx. In this way, yellow fluorescent trypanosomes were recorded for one or more infected proboscides in five experimental crosses (Fig. 2, Additional file 1: Movie S1, Table 2), showing that all three *TCS* strains were mating competent and mating compatible with each of the other strains. Yellow trypanosomes were seen in proboscides from a Gam2 RFP × 1/148 GFP cross as early as 14 days post-infection (dpi) (Additional file 2: Movie S2).

**Table 2:** Summary of five successful interclonal *Trypanosoma congolense* savannah crosses and one intraclonal cross

Parent 1	Parent 2	No. infected proboscides	Fluorescence of trypanosomes in proboscis				
			NF	R	G	RG	RGY
WG81 RFP	1/148 GFP	23	0	12 (52%)	0	10 (44%)^a^	1 (4%)
Gam2 RFP	WG81 GFP	19	4 (21%)	1 (5%)^a^	10 (53%)^b^	1 (5%)	3 (16%)^a,c^
Gam2 RFP	1/148 GFP	33	1 (3%)	0	11 (33%)^a^	11 (33%)	10 (31%)^a,b^
1/148 RFP	WG81 GFP	31	7 (23%)	3 (10%)^a^	10 (32%)^a^	1 (3%)	10 (32%)^a,b^
1/148 RFP	Gam2 GFP	23	5 (22%)	16 (70%)^a^	0	1 (4%)	1 (4%)
1/148 RFP	1/148 GFP	11	2 (18%)	3 (27%)^a^	0	5 (46%)	1 (9%)

NF: non-fluorescent trypanosomes; R: red fluorescent; G: green fluorescent; RG: both red and green fluorescent; RGY: red, green and yellow fluorescent

^a^Non-fluorescent trypanosomes also present

^b^No red fluorescent trypanosomes were seen in two flies

^c^No red fluorescent trypanosomes were seen in one fly

Not all red/green combinations were equally successful in terms of the number of yellow fluorescent hybrids produced, due in part to growth differences between RFP and GFP clones of the same strain. Thus, the outcomes of reciprocal crosses differed; for example, in the Gam2 RFP × 1/148 GFP cross, mixed infections of red and green trypanosomes were found in 21 of 33 proboscides, with yellow trypanosomes in 10 of these, while in the reciprocal cross, 1/148 RFP x Gam2 GFP, mixed infections were found in only two of 23 proboscides, with yellow trypanosomes in a single fly (Table 2). However, looking at the overall percentage of proboscides with yellow trypanosomes in both reciprocal crosses, roughly one fifth had yellow trypanosomes (WG81 × 1/148: 11/54 = 20.4%; Gam2 × WG81: 3/19 = 15.8%; Gam2 × 1/148: 11/56 = 19.6%), showing that all three strain combinations worked satisfactorily in experimental crosses (Table 2). This three-way strain compatibility is similar to that demonstrated previously for *T. brucei* [25], indicating that a simple two mating type system does not apply for either trypanosome species.

Most of the intraclonal crosses attempted were unsatisfactory, because of growth differences between the RFP and GFP clones of the same strain. However, a very low number of yellow fluorescent trypanosomes were found in a single infected proboscis in an intraclonal cross of *TCS* 1/148 (Table 2; Fig. 2G).

A relatively large number of proboscides had only non-fluorescent trypanosomes (19/117 = 16.2%; Table 2), probably because the tsetse alimentary tract was a non-antibiotic-selective environment and trypanosomes that lost expression of the fluorescent transgene had a growth advantage. On the other hand, non-fluorescent hybrids, as well as red, green and yellow fluorescent hybrids, are potential products of sexual reproduction, so we cannot rule out the possibility that some non-fluorescent trypanosomes were actually hybrids.

### Life cycle stage

**Fig. 3:**
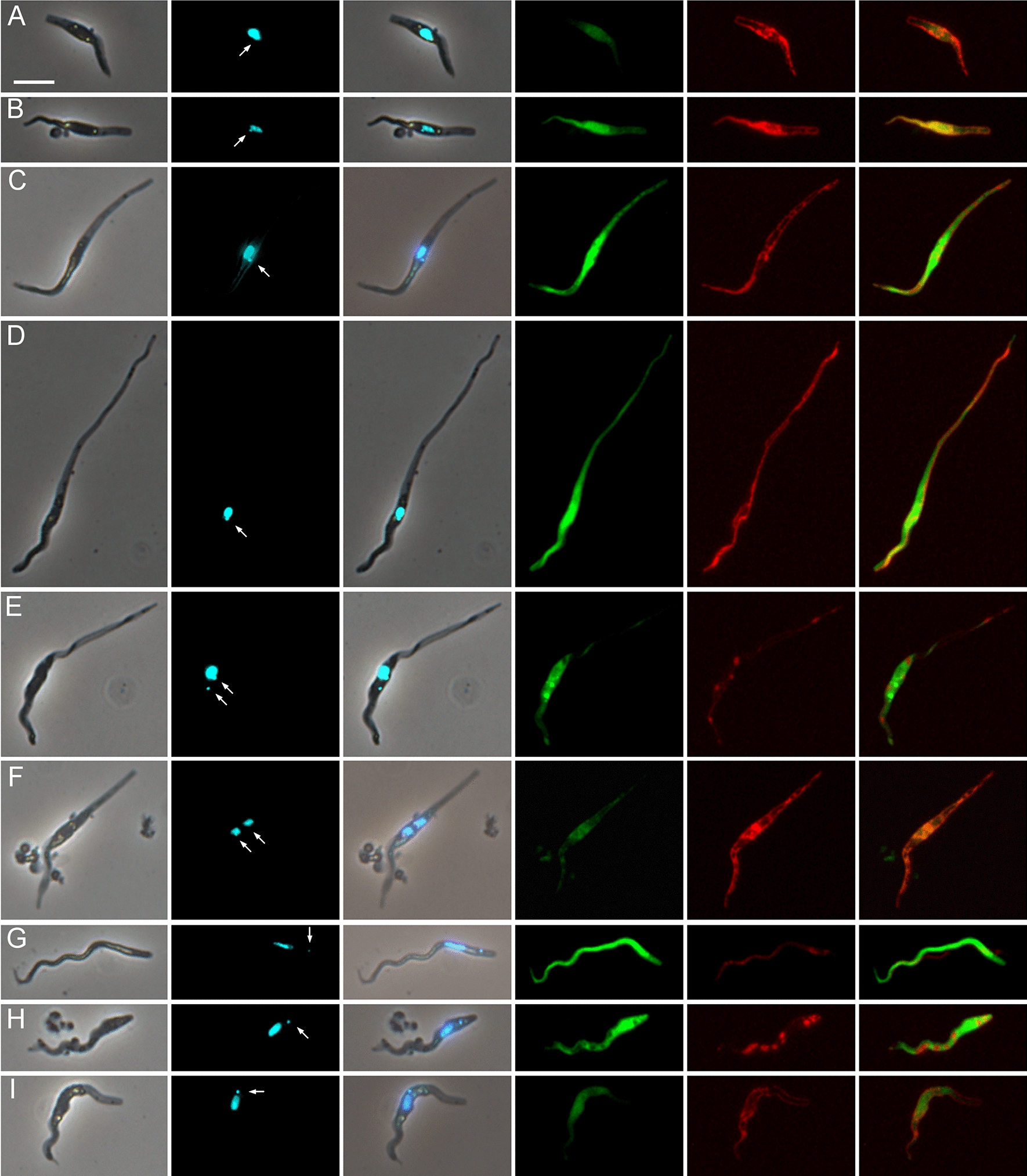
Morphology of yellow fluorescent *TCS* epimastigotes and trypomastigotes from proboscides**.** Examples of yellow fluorescent trypanosomes from proboscides of tsetse infected with Gam2 RFP and 1/148 GFP (dissected 28 days post-infection) and stained live with Hoechst 33342. Epimastigotes (**A**–**F**) varied greatly in morphology; some cells were replicating as they had two kinetoplasts and one or two nuclei (**E**, **F**). Trypomastigotes (**G**–**I**) were usually long and often had pronounced undulations at the anterior end. From left to right: phase contrast, Hoechst 33342, merge, green fluorescence, red fluorescence, merge of red and green fluorescence. Arrows indicate kinetoplasts. Scale bar = 10 µm

To investigate the life cycle stage(s) involved in genetic exchange, trypanosomes from a Gam2 RFP × 1/148 GFP cross were stained with the DNA-binding dye, Hoechst 33342, to visualise the nucleus and kinetoplast in live cells. Several yellow fluorescent trypanosomes were found and these had either epimastigote morphology, with the kinetoplast anterior or juxtaposed to the nucleus (Fig. 3A–F), or trypomastigote morphology, with the kinetoplast posterior to the nucleus (Fig. 3G–I). Epimastigotes varied greatly in length, some having extremely long posteriors (Fig. 3D, E), while others were short (Fig. 3A, B) compared to the more typical length (Fig. 3C). Most trypomastigotes were long with pronounced undulations of the anterior half (e.g. Fig. 3G) and their movement was serpentine (Additional file 3: Movie S3). As these stages are found in the labrum of the proboscis, we infer that hybrid formation occurs prior to invasion of the hypopharynx. No metacyclics were identified among the yellow fluorescent trypomastigotes, probably because these are formed in the hypopharynx and trypanosomes did not readily emerge from this narrow tube when the proboscis was disrupted.

**Fig. 4:**
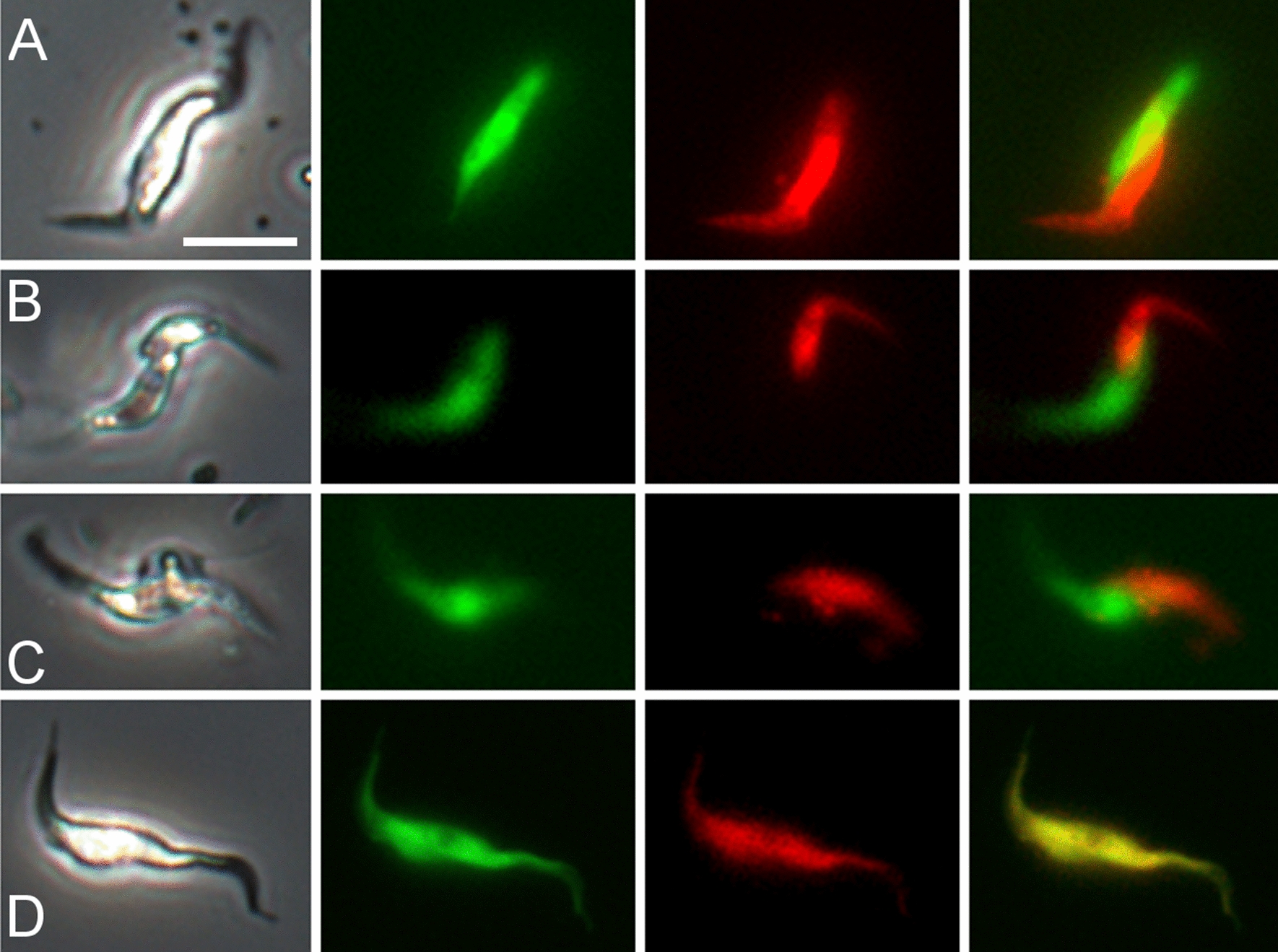
Interacting red and green fluorescent trypanosomes. Trypanosomes from proboscides of tsetse infected with the *TCS* cross Gam2 RFP and 1/148 GFP (dissected 28 days post-infection). Panels **A** - **C** show examples of red and green fluorescent trypanosomes in close contact; the interacting cells are clearly of different sizes in **B**. In Panel **D** the trypanosome appears to have two anterior ends, suggesting recent cell fusion and exchange of cytoplasm. From left to right: phase contrast, green fluorescence, red fluorescence, merge of red and green fluorescence. Scale bar = 10 µm

Red and green trypanosomes were sometimes seen in close contact (Fig. 4A–C; Additional file 4: Movie S4, Additional file 5: Movie S5). Such interactions were observed previously in *T. brucei* crosses of red and green fluorescent trypanosomes [11]. Here, the interacting partners were sometimes clearly of different sizes (e.g. Figure 4B; Additional file 5: Movie S5); however, none of the larger trypanosomes were as long as the epimastigotes described above with an extremely elongated posterior (Fig. 3D). It is tempting to speculate that the cell shown in Fig. 4D, which appears to have two anterior ends, is the result of fusion and merging of cytoplasm.

**Fig. 5:**
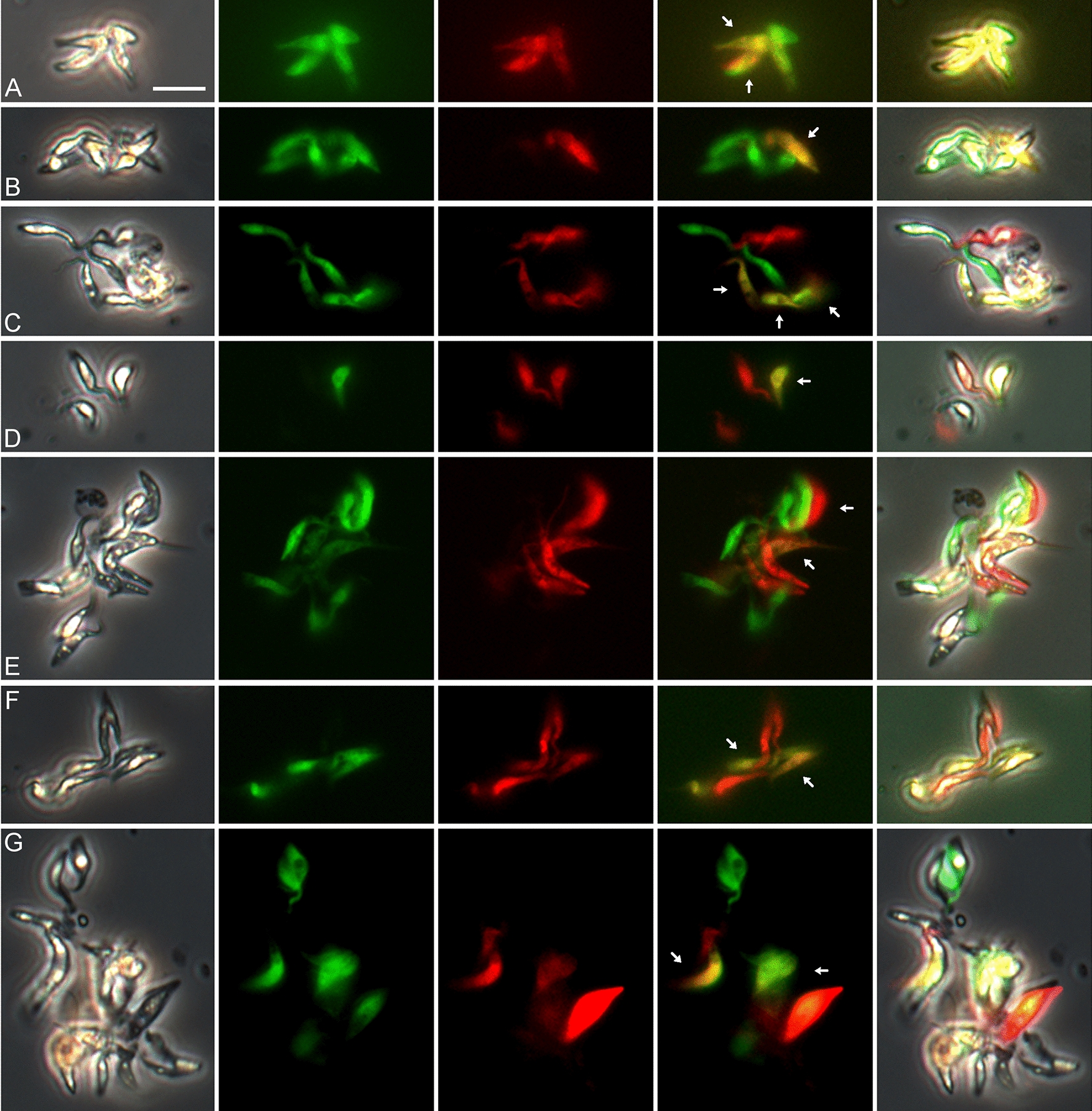
Clumps of red, green and yellow fluorescent trypanosomes. Trypanosomes from proboscides of tsetse infected with the *TCS* cross Gam2 RFP and 1/148 GFP (dissected 32–39 days post-infection). Panels A - G show groups of red, green and yellow fluorescent trypanosomes, which are mostly of short conformation. From left to right: phase contrast, green fluorescence, red fluorescence, merge of red and green fluorescence, merge all. Yellow fluorescent trypanosomes are arrowed. Scale bar = 10 µm

**Fig. 6:**
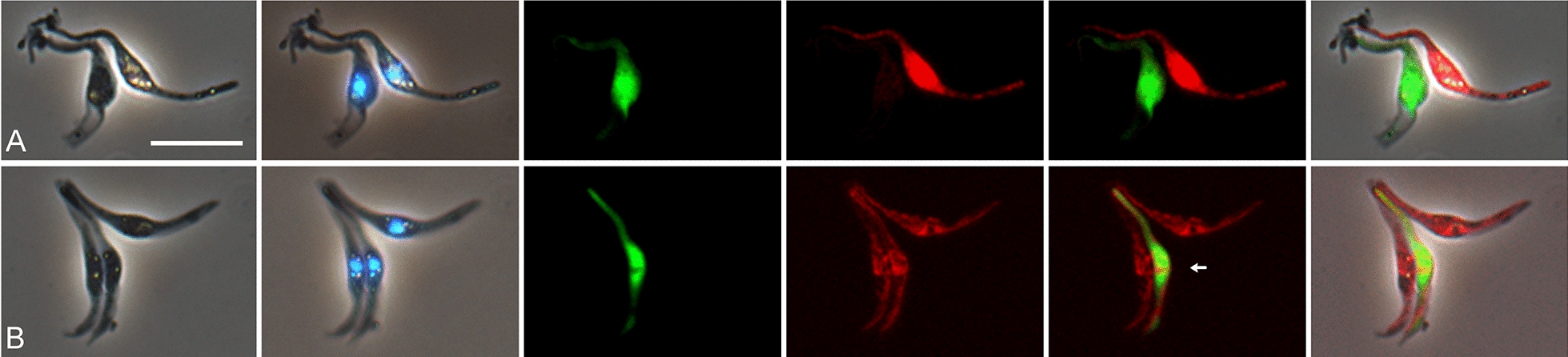
Groups of red, green and yellow fluorescent trypanosomes. Trypanosomes from proboscides of tsetse infected with the *TCS* cross Gam2 RFP and 1/148 GFP (dissected 28 days post-infection). In **A** one red and one green fluorescent trypanosome are adjacent and appear to be attached together at their anterior ends by debris. In **B** one yellow fluorescent trypanosome (arrowed) appears joined to two red ones at the anterior. From left to right: phase contrast, Hoechst 33342 merge, green fluorescence, red fluorescence, merge of red and green fluorescence, merge with phase contrast image. Scale bar = 10 µm

In *T. brucei*, groups of red, green and yellow fluorescent trypanosomes formed after in vitro mixing of salivary-gland-derived cells and these clumps contained many gametes with the characteristic morphology of a pear-shaped body and relatively long flagellum [11]. Similarly, groups of red, green and yellow fluorescent trypanosomes were observed in *TCS* from dissected proboscides (Fig. 5; Additional file 6: Movie S6). The trypanosomes in the *TCS* clumps were mostly of short conformation, but without the long anterior flagellum typical of the *T. brucei* gamete. At higher magnification and stained with Hoechst 33342, these trypanosomes were seen to be short epimastigotes with an elongated posterior, held together at their anterior tips, suggesting recent detachment from a surface (Fig. 6). These clumps resemble the rosettes of epimastigotes attached to the labrum of infected proboscides. If some of these trypanosomes are *TCS* gametes, then gametes in *TCS* and *T. brucei* differ markedly in morphology.

**Fig. 7:**
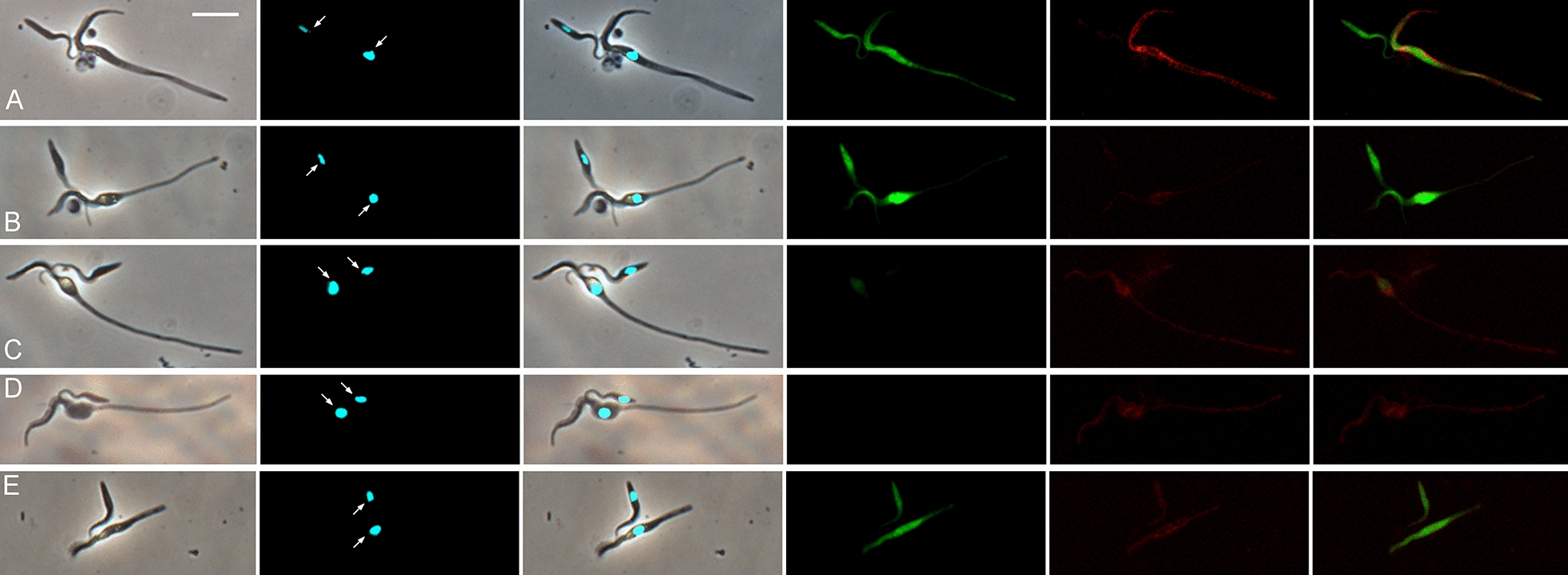
Putative asymmetric division of epimastigotes. Trypanosomes from *TCS* cross Gam2 RFP and 1/148 GFP (dissected 20 days post-infection). Trypanosomes have both red and green fluorescence (**A**, **C** and **E**), but either green (**B**) or red fluorescence (**D**), respectively. From left to right: phase contrast, Hoechst 33,342 merge, green fluorescence, red fluorescence, merge of red and green fluorescence, merge with phase contrast image. Scale bar = 10 µm

One further observation that needs to be mentioned, though it may or may not be relevant in the context of *TCS* mating, is the juxtaposition of very short and very long epimastigotes in what appears to be an asymmetric division, but might also be interpreted as an interaction between a long and short epimastigote (Fig. 7; Additional file 7: Movie S7). Interpretation of the colour of fluorescence of these putative asymmetric dividers is complicated as they were derived from a mixed infection of Gam2 RFP and 1/148 GFP. While some have dual fluorescence (Fig. 7A, C, E), others are either green or red fluorescent (Fig. 7B and D respectively). While the asymmetric division could produce the short epimastigotes that are suggested to be gametes above, the current data are too limited and contradictory to draw any conclusions.

### In vitro mixing of TCS epimastigote cultures

**Fig. 8:**
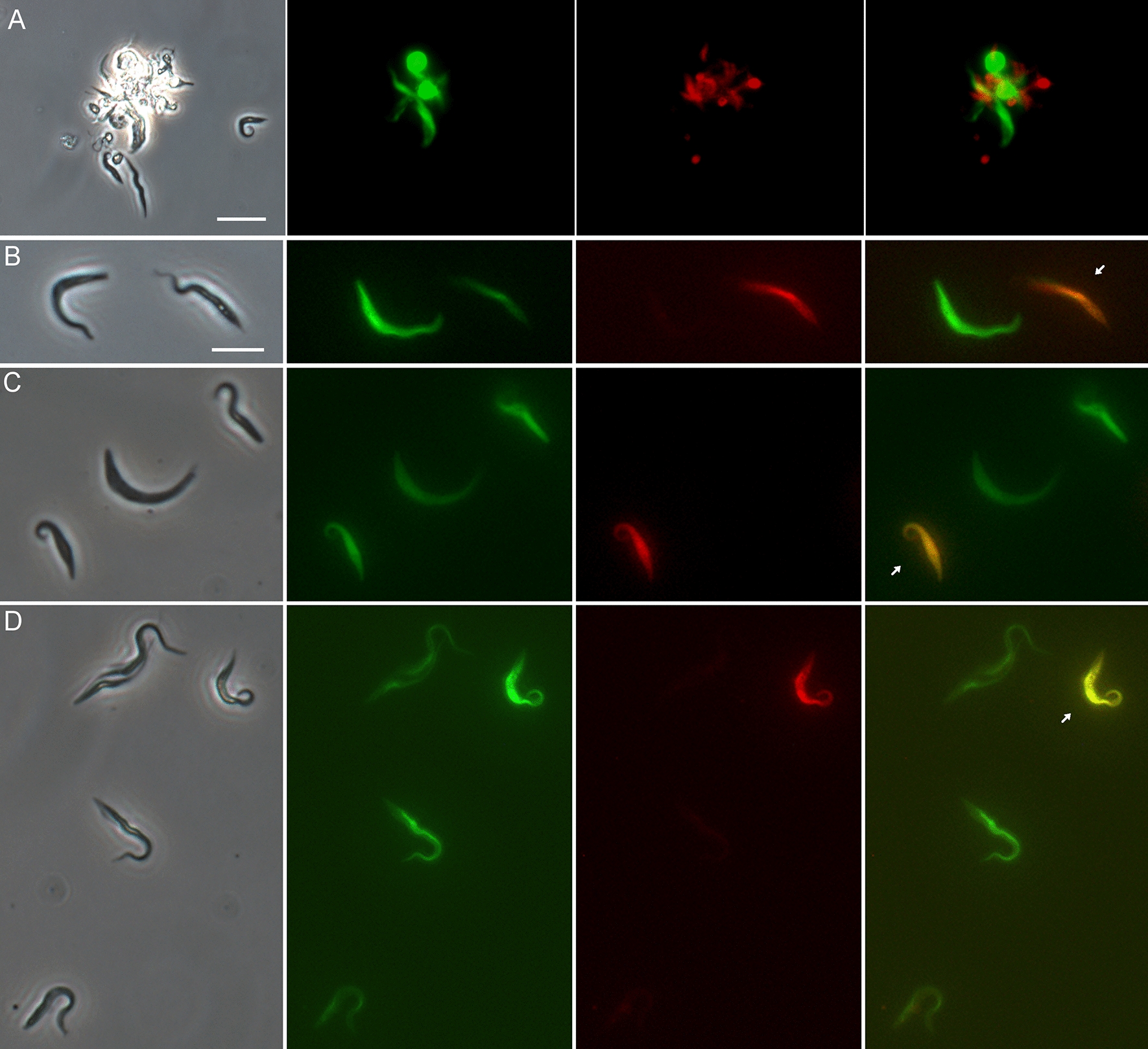
In vitro mixing of red and green fluorescent epimastigotes; 1/148 RFP and Gam2 GFP epimastigotes were mixed in vitro, but very few groups of red and green trypanosomes such as that shown in **A** were found, most clumps being a single colour. Co-cultures were found to contain a few yellow fluorescent trypanosomes (arrowed) at 21 days post-mixing (**B**–**D**), presumably resulting from cytoplasmic exchange between cells of the different strains. From left to right: phase contrast, green fluorescence, red fluorescence, merge of red and green fluorescence. **A** Scale bar = 20 µm; **B**–**D** scale bar = 10 µm

To investigate whether clumps of gametes would form in vitro, we mixed 1/148 RFP and Gam2 GFP, both of which had spontaneously differentiated into attached epimastigotes in long term procyclic culture. Clumps of cells from each epimastigote culture were dislodged, mixed in approximately equal numbers and transferred to individual 1-ml wells, allowing the co-culture to be destructively sampled at intervals (*T* = 1, 3, 5, 6, 21, 28 days). Although red and green fluorescent trypanosomes were present in the co-cultures, they tended to remain in groups of one colour, presumably where the first founder cell had settled, and very few groups of mixed colour were observed (Fig. 8A). Nevertheless, after extensive searching of coverslips by fluorescence microscopy, yellow fluorescent trypanosomes were detected on days 3, 21 and 28 (Fig. 8B–D; Additional file 8: Movie S8); numbers were too low to recover the putative yellow fluorescent hybrids for further analysis.

## Discussion

To date *T. brucei* is the only tsetse-transmitted trypanosome for which sexual reproduction, characterised by meiosis and production of haploid gametes, has been demonstrated experimentally [10–12]. Sexual reproduction takes place in the tsetse salivary glands, most frequently during early establishment of infection [9]. By analogy, we expected that the closely-related trypanosome, *T. congolense* savannah (*TCS*), would also mate during its developmental cycle in the tsetse fly and produce hybrids in the fly proboscis, where the equivalent epimastigote life cycle stages are formed. In the experimental crosses of red and green fluorescent trypanosomes of three different *TCS* strains conducted here, both intra- and interclonal crosses yielded yellow fluorescent hybrids among trypanosomes in the proboscis. However, we were unable to recover individual hybrid trypanosomes for genotyping analysis to confirm inheritance of genes from both parents, as was done extensively for characterisation of *T. brucei* hybrids [9, 25–28]. For *TCS*, the number of trypanosomes in the proboscis was low and the number of yellow fluorescent hybrids even lower, leading to dominance of single colours (i.e. parental trypanosomes) among clones recovered.

Our evidence that the yellow fluorescent trypanosomes are products of the biological process of mating rather than cytoplasmic exchange of proteins [29] rests on three points. First, yellow fluorescent hybrids were found only among trypanosomes isolated from the proboscis, and not from the proventriculus or midgut, despite both these parts of the alimentary tract containing dense populations of red and green fluorescent trypanosomes. Second, this pattern of hybrid occurrence was produced in five different interclonal crosses and one intraclonal cross. Third, replicating yellow fluorescent trypanosomes were observed in vivo, although we were not able to isolate these for further analysis. After cytoplasmic exchange, dual red and green fluorescence was soon lost by turnover of the transferred proteins in a few days [29].

Our search for the specific life cycle stages involved in sexual reproduction such as gametes was inconclusive. Both yellow fluorescent epimastigotes and trypomastigotes were found in the proboscis, but the sequence of occurrence of these individual morphological forms during the life cycle is not known, other than in broad terms. It is known that long proventricular trypomastigotes migrate anteriorly to the fly mouthparts, where they are assumed to attach and differentiate into epimastigotes, which then proliferate [14, 30]. However, the order of occurrence of long and short epimastigotes, and whether morphology and function are linked, are uncertain. Similarly, the stage that invades the hypopharynx and subsequently differentiates into infective metacyclics is not known. Nevertheless, judging from the morphology and location of yellow fluorescent hybrids observed here, the occurrence of sexual reproduction in *TCS* can be narrowed down to stages after proventricular trypomastigotes and before invasion of the hypopharynx. The observation of clumps of red, green and yellow fluorescent trypanosomes was intriguing and may implicate short, attached epimastigotes as the gamete form, in which case *TCS* and *T. brucei* gametes do not share the same morphology. In *T. brucei* the haploid gametes are free-swimming cells with a small pear-shaped body and relatively long flagellum [11]. *TCS* and *T. brucei* gametes form in very different environments: *TCS* gametes have to resist the strong current of blood flow through the feeding canal of the proboscis, whereas *T. brucei* gametes have only peristaltic contractions of the salivary glands to contend with. This biological difference may drive the need for attachment of *TCS* gametes to the walls of the labrum to avoid being washed away and have any chance of mating.

Not all crosses were equally successful in terms of numbers of yellow fluorescent hybrids produced, due in part to differences in growth between red and green fluorescent clones of the same *TCS* strain that affected their ability to compete successfully within the infected fly. As the original *TCS* strains were uncloned, it is possible that heterogeneity within the population led to growth differences in the genetically modified lines. It is also possible that fly transmission of the original strains led to heterogeneity, as cryptic selfing was revealed only by genomic sequencing of *T. brucei* clones [31].

## Conclusions

Yellow fluorescent hybrids were produced after co-transmission of red and green fluorescent lines of *T. congolense* savannah through tsetse flies. The putative hybrids were found only in the tsetse proboscis and not in other parts of the alimentary tract, although the proventriculus and midgut contained dense populations of red and green fluorescent trypanosomes. Both inter- and intraclonal crosses were successful. The three-way strain compatibility observed is similar to that demonstrated previously for *T. brucei* [25], indicating that a simple two mating type system does not apply for either trypanosome species.

### Supplementary Information

**Additional file 1: Movie S1.** Three yellow fluorescent trypanosomes from a Gam2 RFP × 1/148 GFP cross 37–39 days post-infection. Two of the trypanosomes are attached by their posterior tips and are presumed to be in the final stage of cell division.

**Additional file 2: Movie S2.** Yellow fluorescent trypanosome from a Gam2 RFP × 1/148 GFP cross 14 days post-infection. The undulating movement of the cell anterior is presumably caused by a flagellum too closely opposed to the cell body to be visible, while the flagellum projecting from the cell body suggests either that this is a replicating cell or a recent fusion of two trypanosomes.

**Additional file 3: Movie S3.** Serpentine movement of a yellow fluorescent trypomastigote from a Gam2 RFP × 1/148 GFP cross 28 days post-infection. In the final frames, Hoechst 33342 fluorescence reveals the kinetoplast and nucleus.

**Additional file 4: Movie S4.** Interacting red and green trypanosomes from a Gam2 RFP × 1/148 GFP cross at 28 days post-infection. The red and green cells are of approximately equal size.

**Additional file 5: Movie S5.** Interacting red and green trypanosomes from a WG81 RFP × 1/148 GFP cross at 24 days post-infection. The smaller trypanosome is red and the larger is green.

**Additional file 6: Movie S6.** Clump of green and yellow fluorescent trypanosomes from a Gam2 RFP × 1/148 GFP cross 37–39 days post-infection.

**Additional file 7: Movie S7.** Asymmetric division of epimastigotes. Gam2 RFP 1/148 GFP 20 days.

**Additional file 8: Movie S8** Two yellow fluorescent trypanosomes from an in vitro epimastigote cross of 1/148 RFP × Gam2 GFP cross at 21 days.

## Data Availability

All data generated or analysed during this study are included in this published article and its supplementary information.

## Abbreviations

CM: Cunningham’s medium
dpi: Days post-infection
FCS: Foetal calf serum
GFP: Green fluorescent protein
PBS: Phosphate-buffered saline
RFP: Red fluorescent protein
TCS: *Trypanosoma congolense* savannah

## References

[1] Programme Against African Trypanosomosis (PAAT). http://www.fao.org/ag/againfo/programmes/en/paat/disease.html.

[2] Shaw APM, Cecchi G, Wint GRW, Mattioli RC, Robinson TP (2014). Mapping the economic benefits to livestock keepers from intervening against bovine trypanosomosis in Eastern Africa. Prev Vet Med.

[3] Auty H, Torr SJ, Michoel T, Jayaraman J, Morrison LJ (2015). Cattle trypanosomosis: the diversity of trypanosomes and implications for disease epidemiology and control. Rev Sci Tech Off Int Epiz.

[4] Morrison LJ, Vezza L, Rowan T, Hope JC (2016). Animal African Trypanosomiasis: time to increase focus on clinically relevant parasite and host species. Trends Parasitol.

[5] African Union: Interafrican Bureau for Animal Resources. http://www.au-ibar.org/index.php?option=com_flexicontent&view=items&cid=57&id=63&Itemid=37&lang=en. 10.1136/vr.d7271 22081643

[6] Gibson W (2007). Resolution of the species problem in African trypanosomes. Int J Parasitol.

[7] Bengaly Z, Sidibe I, Ganaba R, Desquesnes M, Holby H, Sawadogo L (2002). Comparative pathogenicity of three genetically distinct types of *Trypanosoma congolense* in cattle: clinical observations and haematological changes. Vet Parasitol.

[8] Tihon E, Imamura H, Dujardin JC, Van Den Abbeele J, Van den Broeck F (2017). Discovery and genomic analyses of hybridization between divergent lineages of *Trypanosoma congolense*, causative agent of Animal African Trypanosomiasis. Mol Ecol.

[9] Gibson W, Peacock L, Ferris V, Williams K, Bailey M (2008). The use of yellow fluorescent hybrids to indicate mating in *Trypanosoma brucei*. Parasit Vectors.

[10] Peacock L, Ferris V, Sharma R, Sunter J, Bailey M, Carrington M, Gibson W (2011). Identification of the meiotic life cycle stage of *Trypanosoma brucei* in the tsetse fly. Proc Natl Acad Sci USA.

[11] Peacock L, Bailey M, Carrington M, Gibson W (2014). Meiosis and haploid gametes in the pathogen *Trypanosoma brucei*. Curr Biol.

[12] Peacock L, Kay C, Farren C, Bailey M, Carrington M, Gibson W (2021). Sequential production of gametes during meiosis in trypanosomes. Commun Biol.

[13] Morrison LJ, Tweedie A, Black A, Pinchbeck GL, Christley RM, Schoenefeld A, Hertz-Fowler C, MacLeod A, Turner CMR, Tait A (2009). Discovery of mating in the major African livestock pathogen *Trypanosoma congolense*. PLoS ONE.

[14] Peacock L, Cook S, Ferris V, Bailey M, Gibson W (2012). The life cycle of
* Trypanosoma (Nannomonas) congolense
* in the tsetse fly. Parasit Vectors.

[15] Peacock L, Kay C, Collett C, Bailey M, Gibson W (2023). Development of the livestock pathogen
* Trypanosoma (Nannomonas) simiae
* in the tsetse fly with description of putative sexual stages from the proboscis. Parasit Vectors.

[16] Peacock L, Ferris V, Bailey M, Gibson W (2008). Fly transmission and mating of *Trypanosoma brucei brucei* strain 427. Mol Biochem Parasitol.

[17] Peacock L, Ferris V, Bailey M, Gibson W (2009). Intraclonal mating occurs during tsetse transmission of *Trypanosoma brucei*. Parasit Vectors.

[18] Cunningham I (1977). New culture medium for maintenance of tsetse tissues and growth of trypanosomatids. J Protozool.

[19] Kay C, Peacock L, Gibson W (2019). *Trypanosoma congolense*: in vitro culture and transfection. Curr Protoc Microbiol.

[20] Bingle LEH, Eastlake JL, Bailey M, Gibson WC (2001). A novel GFP approach for the analysis of genetic exchange in trypanosomes allowing the in situ detection of mating events. Microbiology.

[21] Downey N, Donelson JE (1999). Search for promoters for the GARP and rRNA genes of *Trypanosoma congolense*. Mol Biochem Parasitol.

[22] Galun R, Margalit J (1969). Adenine nucleotides as feeding stimulants of tsetse fly *Glossina austeni* Newst. Nature.

[23] Macleod ET, Maudlin I, Darby AC, Welburn SC (2007). Antioxidants promote establishment of trypanosome infections in tsetse. Parasitology.

[24] Maser P, Grether-Buhler Y, Kaminsky R, Brun R (2002). An anti-contamination cocktail for the in vitro isolation and cultivation of parasitic protozoa. Parasitol Res.

[25] Turner CMR, Sternberg J, Buchanan N, Smith E, Hide G, Tait A (1990). Evidence that the mechanism of gene exchange in *Trypanosoma brucei* involves meiosis and syngamy. Parasitology.

[26] Jenni L, Marti S, Schweizer J, Betschart B, Lepage RWF, Wells JM, Tait A, Paindavoine P, Pays E, Steinert M (1986). Hybrid formation between African trypanosomes during cyclical transmission. Nature.

[27] Paindavoine P, Zampetti-Bosseler F, Pays E, Schweizer J, Guyaux M, Jenni L, Steinert M (1986). Trypanosome hybrids generated in tsetse flies by nuclear fusion. EMBO J.

[28] MacLeod A, Tweedie A, McLellan S, Taylor S, Cooper A, Sweeney L, Turner CMR, Tait A (2005). Allelic segregation and independent assortment in *Trypanosoma brucei* crosses: proof that the genetic system is Mendelian and involves meiosis. Mol Biochem Parasitol.

[29] Imhof S, Fragoso C, Hemphill A, von Schubert C, Li D, Legant W, Betzig E, Roditi I (2016). Flagellar membrane fusion and protein exchange in trypanosomes; a new form of cell-cell communication?. F1000 Res.

[30] Peacock L, Kay C, Bailey M, Gibson W (2018). Shape-shifting trypanosomes: Flagellar shortening followed by asymmetric division in *Trypanosoma congolense* from the tsetse proventriculus. PLoS Path.

[31] Kay C, Peacock L, Williams TA, Gibson W (2022). Signatures of hybridization in *Trypanosoma brucei*. PLoS Path.

